# Anatomy of the Horse

**Published:** 1884-10

**Authors:** 


ANATOMY OF THE HORSE.

We have seen a few pages of the advance sheets of a new veterinary book on the anatomy of the horse, by Professor McFadyean, of the Royal Veterinary College of Edinburgh. The work will consist of 350 pages, and will contain 48 full colored lithographed plates, and 40 cuts in the text. It will be published by W. R. Jenkins, and the price will be about $5. The want of such a work has long been felt, and the price being so moderate we have no doubt that every veterinarian will procure a copy.

